# Insight into Lotusine and Puerarin in Repairing Alcohol-Induced Metabolic Disorder Based on UPLC-MS/MS

**DOI:** 10.3390/ijms231810385

**Published:** 2022-09-08

**Authors:** Jiayang Xu, Xiaoyue Zhang, Lili Yan, Zhichao Zhang, Jing Wei, Luqi Li, Qiang Zhang

**Affiliations:** 1 Shaanxi Key Laboratory of Natural Products & Chemical Biology, College of Chemistry & Pharmacy, Northwest A&F University, Yangling, Xianyang 712100, China; 2 College of Biology Pharmacy & Food Engineering, Shangluo University, Shangluo 726000, China; 3 Life Science Research Core Services, Northwest A&F University, Yangling, Xianyang 712100, China

**Keywords:** widely targeted metabolome, metabolic disease, alcoholism, LC-ESI-MS, lotusine, puerarin

## Abstract

Alcohol is an essential element in human culture. However, alcoholism has contributed to numerous health issues, including alcoholic fatty liver and sudden death. We found that the alkaloid lotusine possessed hepato- and neuroprotection against alcohol injuries. Lotusine showed comparable protective effects to puerarin, a widely recognized antagonist against alcohol damage. To better understand the metabolic response to alcohol injury and antagonist molecules, we applied sensitive zebrafish and LC-ESI-MS to collect metabolites related to alcohol, puerarin and lotusine exposure. LC-MS identified 119 metabolites with important physiological roles. Differential metabolomic analysis showed that alcohol caused abnormal expression of 82 metabolites (60 up-regulated and 22 down-regulated). These differential metabolites involved 18 metabolic pathways and modules, including apoptosis, necroptosis, nucleotide and fatty acid metabolism. Puerarin reversed seven metabolite variations induced by alcohol, which were related to necroptosis and sphingolipid metabolism. Lotusine was found to repair five metabolites disorders invoked by alcohol, mainly through nucleotide metabolism and glutathione metabolism. In phenotypic bioassay, lotusine showed similar activities to puerarin in alleviating behavioral abnormalities, neuroapoptosis and hepatic lipid accumulation induced by alcohol exposure. Our findings provided a new antagonist, lotusine, for alcohol-induced damage and explored the roles in repairing abnormal metabolism.

## 1. Introduction

Liquor plays an important role in human culture, but excessive drinking causes serious physical, psychological, and social problems. According to the World Health Organization (WHO) statistics, about 3 million people died from drinking in 2016, accounting for 53% of all deaths worldwide, which has caused an enormous burden on global public health [1,2]. The consumption level of alcohol will continue to increase in the next few years, and the related hazards will follow. Excessive drinking can quickly induce body dysfunction, even premature death [3]. For example, alcoholic liver disease is the main cause of chronic liver disease [4,5]. Fatty liver is the early stage of alcoholic liver disease [6], mainly manifested in the liver’s excessive deposition of triglycerides, phospholipids and cholesterol esters [7]. However, the increasingly toxic side effects of alcohol and its metabolites on the liver eventually lead to alcoholic cirrhosis. In most cases, this process is irreversible [8]. Long-term drinking will injure the central and peripheral nervous system, resulting in cognitive impairment, slow response, limb numbness, etc. [9,10]. Excessive drinking is also the most common cause of alcoholic neuropathy, a persistent disease caused by abnormalities in the central nervous system [11]. Recent studies have shown that in about 25 to 65% of patients long-term drinking will lead to the down-regulation of central nervous system receptors, thereby inducing neuropathy [12].

Alcohol metabolism is one critical factor that can impact alcoholism development [13]. Excessive drinking greatly perturbed lipid metabolome and hepatocyte redox homeostasis [14,15]. Current research on excessive drinking mainly focuses on evaluating pharmacodynamic indicators and profiling the expression variation in genes and proteins. However, the metabolic dysfunction perturbed by alcoholism has been very limitedly investigated.

**Figure 1:**
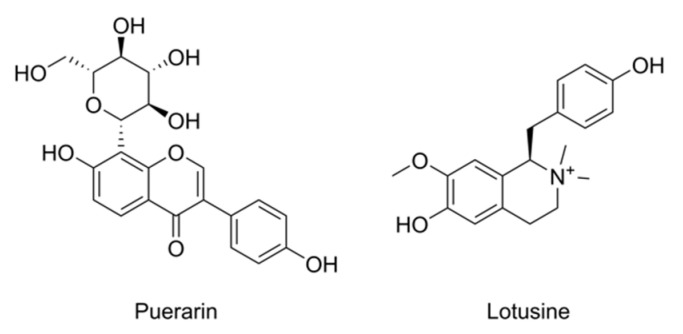
Chemical Structures of puerarin and lotusine.

Natural products provide a promising approach to preventing disease development [16]. The natural product puerarin (Figure 1) can activate the AMPK pathway to relieve hepatocyte autophagy and hepatic lipid deposition induced by alcohol [17,18,19]. Puerarin also protects against alcohol-induced injuries to the central nervous by reducing the activation of microglia [20]. In our bioactivity screening based on metabolic analysis, we found that the natural product lotusine (Figure 1) can alleviate the damage invoked by alcoholism. The compound lotusine is a typical alkaloid from the traditional Chinese herb medicine *Nelumbinis plumula*, which is a dried leaflet and radicle in the mature seeds of a lotus (*Nelumbo nucifera* Gaertn.) [21]. Few studies have been reported on the biological activity of the benzyltetrahydroisoquinoline alkaloid lotusine. It has potential cardiac protective activity against doxorubicin-induced oxidative stress [22] and alleviates UV-induced expression of MMP-1 in skin keratinocytes, thus having potential anti-wrinkle effects [23].

Herein, we investigated the repair effect of the two compounds (puerarin and lotusine) on metabolic abnormalities caused by alcohol and provided potential biomarkers to resist the adverse effects of excessive alcohol intake.

We selected zebrafish as an insensitive platform to investigate metabolic variation. Zebrafish, a small tropical freshwater fish, have been widely used in pharmacology and toxicology in recent years as a model organism with high homology to the human genome [24,25]. In contrast to rodents, zebrafish embryos are transparent, and their major organs are similar to those of mammals. They can be easily observed, which is used to evaluate the acute toxicity, hepatotoxicity, cardiotoxicity, nephrotoxicity, developmental toxicity and neurotoxicity of drugs [26]. The larvae have the advantages of short, individual developmental cycles, similar metabolic pathways in vivo to humans, and require only a trace amount of tested compounds. It has also become the preferred platform to explore organism responses to exogenous stimuli [27,28,29]. Thus, combining the zebrafish model with LC-MS-based metabolic exploration has excellent broad prospects for mining metabolic regulation functions.

## 2. Results

### 2.1. LC-MS/MS Based Metabolism Analysis

**Figure 2:**
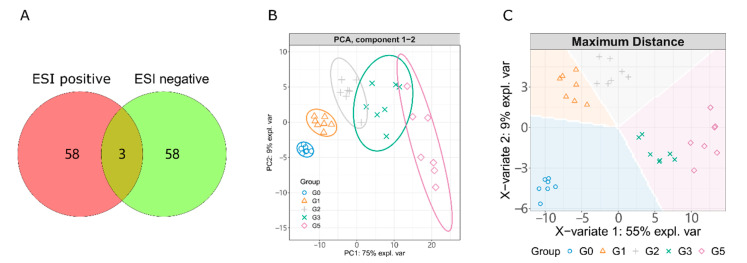
(**A**) Numbers of metabolites identified by positive and negative UPLC-ESI-MS. PCA (**B**) and sPLS-DA (**C**) analysis of LC-MS profiles of metabolism. The colored background represents predicted classes.

Differential metabolomics are commonly used to explore the small molecule markers of disease, biological functions and mechanisms of small molecules [30,31]. To obtain the key difference metabolites, we designed five experimental groups for this purpose, which were the blank group (G0), 1% alcohol exposure group (G1), 2% alcohol exposure group (G2), 40 µM puerarin treated group (G3) and 40 µM lotusine treated group (G5). As shown in Figure 2A and Table S1, a total of 119 metabolites were identified from ESI positive and negative ions. Only three metabolites were identified from both ESI positive and negative resources. Positive and negative ion sources can complement each other in identifying metabolites.

We pooled quantitative information of all samples into an unsupervised principal component analysis (PCA) model and a supervised sparse partial least squares discriminant analysis (sPLS-DA) model. PCA and sPLS-DA can extract inter-sample differences and downscale the multidimensional data under unsupervised and supervised conditions, respectively. As shown in Figure 2B,C, the principal components 1 and 2 (PC1 and PC2) in PCA gathered 84% of the features among samples. sPLS-DA is an unsupervised pattern recognition method with a more robust discriminant function. Each group clustered well and separated from the others, coinciding with the different experimental treatments. The two alcohol exposure groups (G1 and G2) were closer since they were treated with the only different concentrations of alcohol in the experiment. The sPLS-DA feature-group predictions (background color of Figure 2C) indicated that G3 and G5 were closely featured, but the samples in the two groups separately clustered. This related to the same treatment process for both groups and suggested differences between them.

### 2.2. Alcohol Induced Metabolism Variation

**Figure 3:**
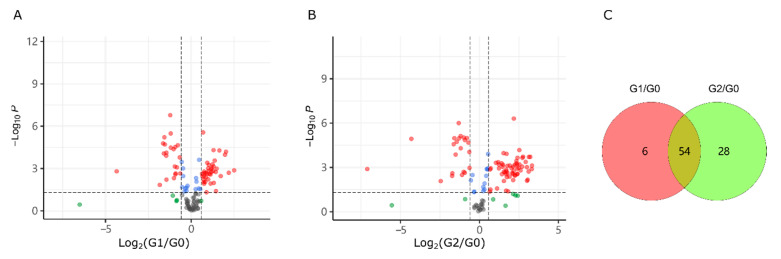
Metabolism variation induced by exposures with 1% (**A**) and 2% alcohol (**B**) and the number of differential metabolites (**C**). G1/G0, G1 vs. G0; G2/G0, G2 vs. G0.

We filtered differential metabolites on the thresholds of FC > 1.5 and statistical *p*-value < 0.05. As shown in Figure 3A,B, high alcohol concentration exposure (2% in G2) caused more various metabolites than that in G1 group (1% alcohol exposed). Details of these differential metabolites were listed in Tables S2 and S3. G1 treatment interfered with the synthesis of 60 metabolites, including 39 up-regulated and 21 down-regulated metabolites. The G2 group (2% alcohol exposure) up-regulated 60 metabolites and down-regulated 22 metabolites. As shown in Figure 3C, the metabolite responses induced by low and high concentrations of alcohol exposure were mostly consistent (54 same differential metabolites) but not identical. In both groups of G1 and G2, the number of up-regulated metabolites was more than that of down-regulated metabolites.

**Figure 4:**
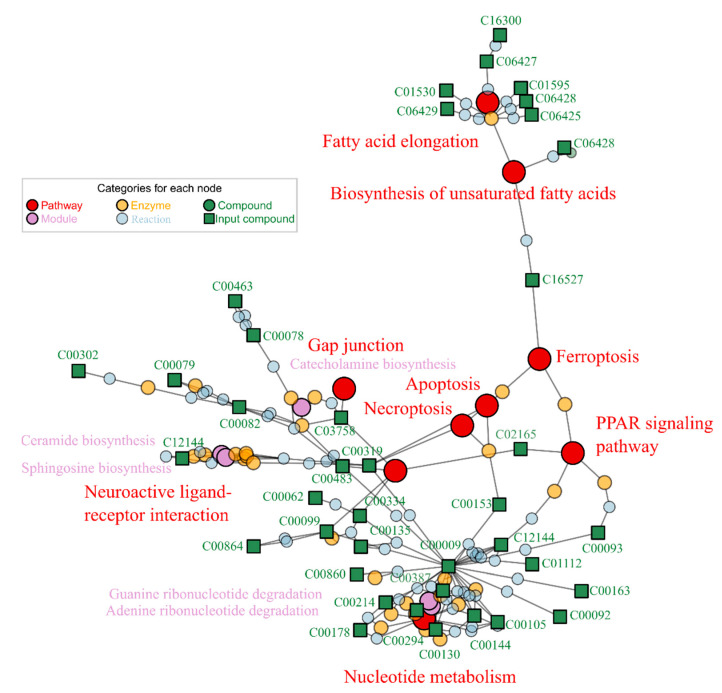
Metabolome response to alcohol exposure (G2) in zebrafish.

The differential metabolites from the two contrasts (G1/G0 and G2/G0) were enriched separately in FELLA for functional annotation (Table 1). Enrichments were calculated through diffusion processes which could annotate pathway and module information to submitted metabolites [32]. We selected those enriched KEGG nodes (pathway, module, enzyme, reaction, etc.) using the threshold of *p* score < 0.01 (generally 0.05). Lower *p* scores (or higher −log (*p*)) indicated higher reliability of enrichment. The enriched KEGG pathways and modules were pooled in Table 1, and the relationship among the KEGG nodes (including pathways, modules, enzymes and reactions) is shown in Figure 4. The metabolic pathways disturbed by 1 and 2% alcohol (G1 and G2) were mostly consistent. G1 and G2 treatments mainly disturbed the pathways of fatty acid elongation, apoptosis, necroptosis and biosynthesis of unsaturated fatty acids, nucleotide metabolism and thiamine metabolism. The main interfered modules were sphingosine biosynthesis, adenine ribonucleotide degradation and ceramide biosynthesis under 1 and 2% alcohol exposures.

Given that more metabolic pathways and modules were observed with G2 exposure, we used the G2 treatment as a representative to explore metabolic damage and repair. The matched differential metabolites and enriched KEGG nodes were mapped in Figure 4. The pathway or module with more metabolite matches indicates a more significant impact. Based on the number of matched metabolites, the most influenced pathways by alcohol were fatty acid elongation, necroptosis, nucleotide metabolism, and neuroactive ligand-receptor interaction. Necroptosis and neuroactive ligand-receptor interaction pathways were at the center of the enrichment map. The metabolite orthophosphate (C00009) on the nucleotide metabolism pathway had the most connections to other nodes and was associated with the phosphorylation process in the pathway. The enriched metabolic network graph can visualize the association among all matching nodes. Limited to the maximum of plotting nodes, not all matching nodes can be presented in Figure 4. Therefore, we summarized the complete enriched pathways and modules in Table 1. In summary, 2% alcohol exposure (G2) affected 12 pathways and six metabolic modules.

### 2.3. Puerarin Repair against Metabolism Disorder Induced by Alcohol

**Figure 5:**
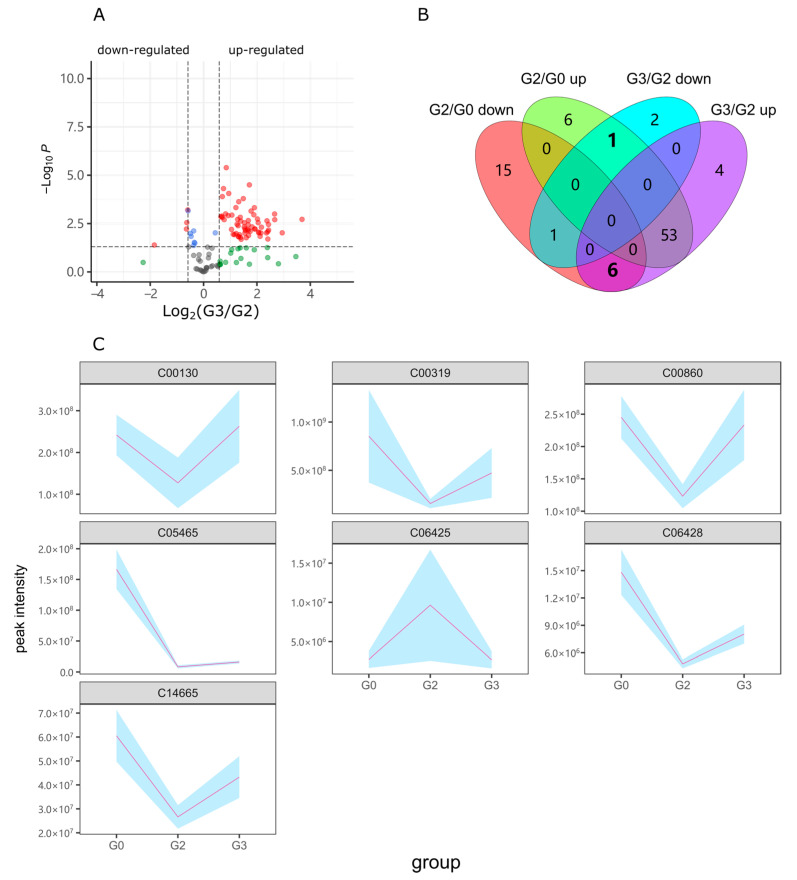
Differential metabolites induced by puerarin. (**A**) Volcano plot of the contrast between G3 and G2. (**B**) Number of changed metabolites compared with G2/G0. (**C**) Response metabolites with reverse changing trends when zebrafish were exposed to alcohol and puerarin. The light blue background represents the standard deviation range. Statistical *p* < 0.05; Fold Change > 1.5; G2/G0, G2 vs. G0; G3/G2, G3 vs. G2.

Group G3 was exposed to 40 µM puerarin and 2% alcohol. The concentration of alcohol was consistent with the G2 group. Thus, when filtering differential metabolites, the G3 group was compared with the G2 group rather than the G1 group. As shown in Figure 5A,B, and Table S4, G3 up-regulated 63 metabolites and down-regulated 4 metabolites compared with G2. Among these differential metabolites, one alcohol-up-regulated metabolite decreased in G3, and six metabolites down-regulated by alcohol increased in G3 (Figure 5B). The total seven metabolites with inverse change were gathered, as shown in Figure 5C, which reflected the reparative effects of puerarin on alcohol metabolic damage.

**Figure 6:**
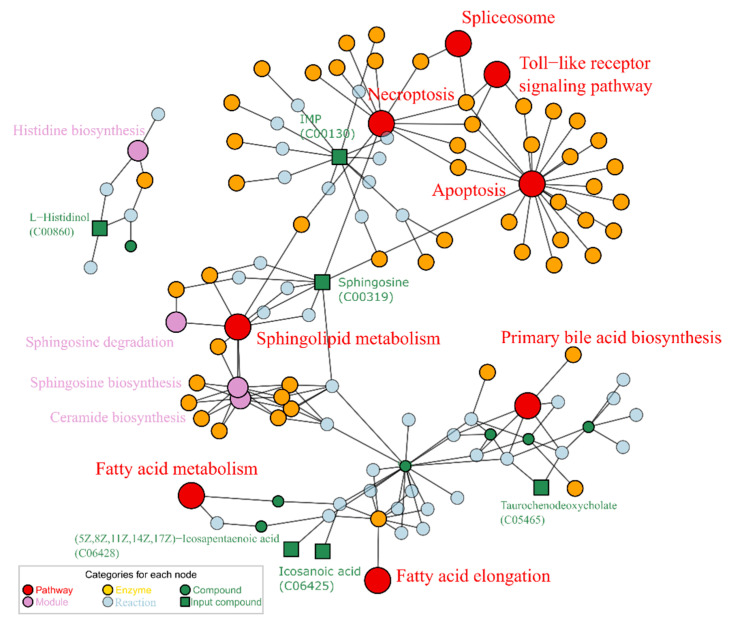
Enriched map based on repaired metabolites by puerarin, enrichment *p* score < 0.01.

All the reversely changed metabolites (7 in total) were enriched into KEGG nodes by FELLA, as shown in Figure 6. Six metabolites, except for the steroid Oranabol (C14665), matched with KEGG pathways. The metabolite IMP (C00130) matched with Necroptosis, which is related to apoptosis, spliceosome, and Toll-like receptor signaling pathways. The compound C00319 connected apoptosis with the sphingolipid metabolism pathway. The compound L-Histidinol matched with the module histidine biosynthesis. The other three metabolites (C05465, C06425, and C06428) were related to fatty acids synthesis and metabolism, which are also associated with the pathway biosynthesis of unsaturated fatty acids. The compound C06428 showed apparent trends of reverse variation among the treatment groups G0, G2, and G3. Not all eligible metabolites can be attributed to a KEGG pathway by the current enrichment algorithm, such as C14665. This might be due to a lack of corresponding pathway information or not meeting the minimum requirements of enrichment.

### 2.4. Lotusine Repair against Metabolism Disorder Induced by Alcohol

**Figure 7:**
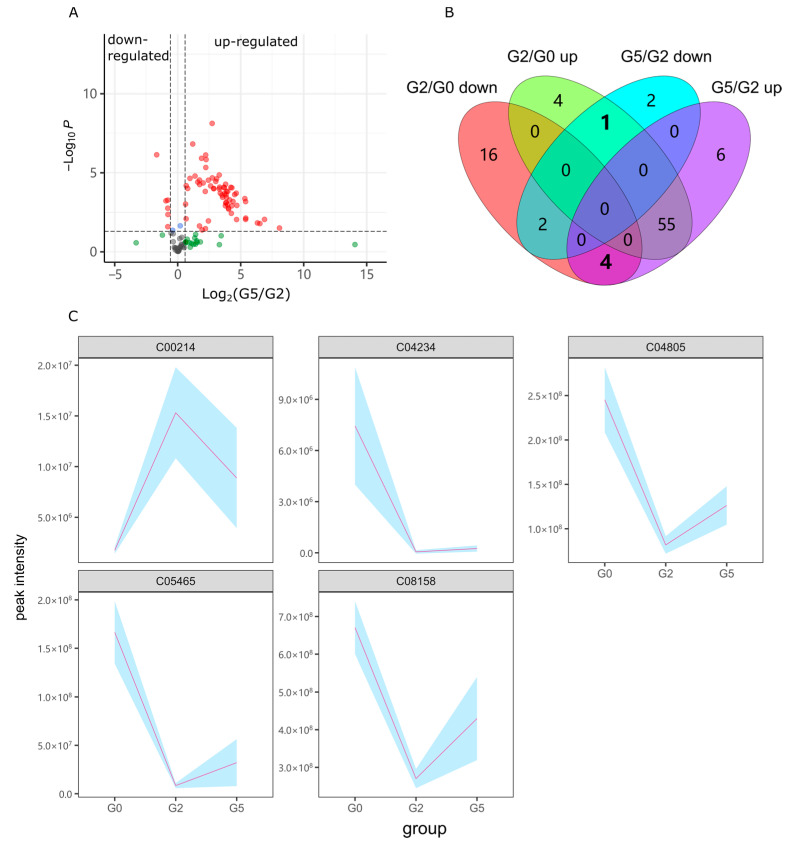
Differential metabolites induced by lotusine. (**A**) Volcano plot of the contrast G5/G2. (**B**) Numbers of changed metabolites compared with G2/G0. (**C**) Response metabolites with reverse changing trends when zebrafish were exposed to alcohol and lotusine. The light blue background represents the standard deviation range. Statistical *p* < 0.05; Fold Change > 1.5; G2/G0, G2 vs. G0, G5/G2, G5 vs. G2.

Group G5 was exposed to 40 µM lotusine and 2% alcohol. The differential metabolites were filtered out based on the contrast of G5 vs. G2. As shown in Figure 7A,B, as well as Table S5, in the contrast of G5/G2, 65 metabolites were up-regulated and 5 metabolites down-regulated. Among these metabolites, 1 alcohol- up-regulated metabolites decreased in G5, and 4 metabolites down-regulated by alcohol increased in G5 (Figure 7B). The total 5 metabolites with inverse change were gathered as shown in Figure 7C. Here two sharply changing metabolites emerged, 2-carboxy-D-arabinitol 1-phosphate (C04234) and taurochenodeoxycholate (C05465). Compared with the blank control G0, C04234 and C06465 dropped to only 0.7% and 5.0% of normal levels in G0 after alcohol exposure (G2). Both metabolites did not restore significantly (*p* > 0.05) with puerarin treatment (G3). But in G5, they recovered to 3.4% and 19.3% of normal levels with lotusine treatment. These were distinct regulatory sites for the metabolic repair function of lotusine different with puerarin.

**Figure 8:**
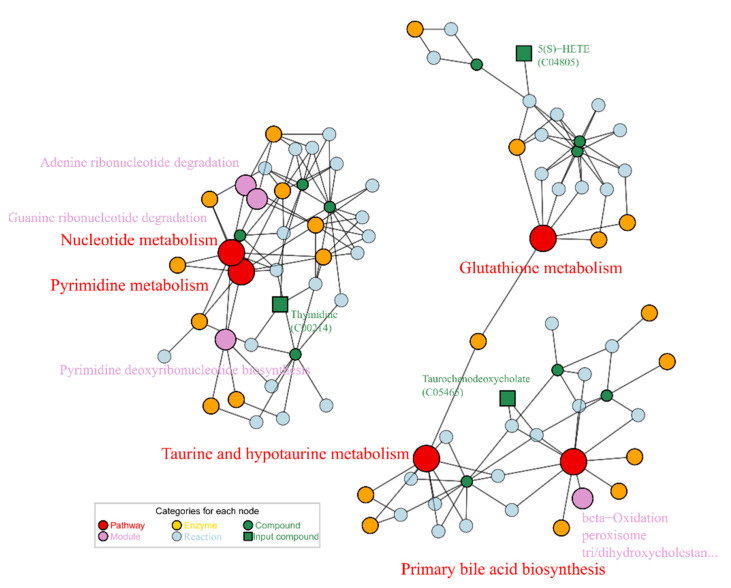
Enriched map based on repaired metabolites by lotusine, enrichment *p* score < 0.01.

Then the 5 metabolites screened out were enriched for KEGG nodes. As showed in Figure 8, the enrichment result afforded 5 related pathways: pyrimidine metabolism (map00240), nucleotide metabolism (map01232), taurine and hypotaurine metabolism (map00430), and glutathione metabolism (map00480). One dramatically changed metabolite C05465 is associated with two pathways: primary bile acid biosynthesis (map00120), and taurine and hypotaurine metabolism (map00430). Another metabolite C04234 was not enriched to any KEGG pathway or modules. The metabolite thymidine (C00214) matched with nucleotide metabolism, which was the mainly affected pathway by alcohol exposure.

### 2.5. Neuroprotective Effects against Alcohol-Induced Damage

Alcohol damage to an organism is mainly on neurological and hepatic impairment. Lotusine is the natural product we have identified as a buffering agent against alcohol injury. Puerarin has been reported to relieve alcohol injury on the liver. In the phenotype verification, we compared the neuroprotective and hepatoprotective effects of both puerarin and lotusine.

**Figure 9:**
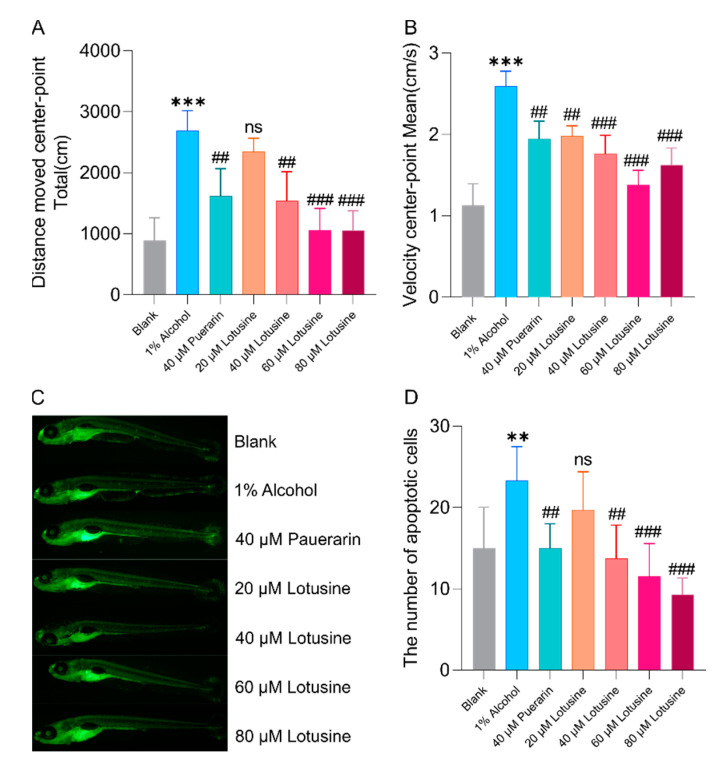
Characterization of the neuroprotective activity of lotusine. (**A**,**B**) zebrafish larvae behavior treated with lotusine. (**C,D**) The effect of lotusine on the neural cells of zebrafish larvae. ** *p* < 0.01 vs. blank; *** *p* < 0.001 vs. blank; ## *p* < 0.01 vs. alcohol exposure group; ### *p* < 0.001 vs. alcohol expose group; ns, not significant; *n* = 7.

Behavioral observation of animals is a standard phenotypic measurement of nerve damage. When the animal nervous system is disturbed or damaged, it is often manifested as abnormal running behavior, including motor acceleration and regional selectivity of movement. The larvae increased swimming distance and velocity when exposed to 1% alcohol for 0.5 h (Figure 9A,B). Pre-treatment with puerarin or lotusine delayed alcohol-induced behavioral abnormalities. As shown in Figure 9A,B, zebrafish larvae showed decreased movement distance and speed in a dose-dependent manner. At the concentration of 40 μM, puerarin and lotusine have comparable effects on moving distance and velocity.

Due to the transparency of zebrafish larvae body, the apoptotic cells in the central nervous system can be observed by fluorescent staining under a fluorescence microscope. After acridine orange staining, the apoptotic cells are displayed as tiny bright spots in green fluorescent, as shown in Figure 9C. The apoptotic cells were quantified using ImageJ, as shown in Figure 9D. Alcohol caused more apoptotic cells (23) than that in blank control (16). When treated with puerarin (40 μM), the apoptotic cell number dropped to a normal level (15). Lotusine showed a dose-dependent manner in reducing apoptosis. The anti-apoptotic effects of lotusine and puerarin at the same concentrations (40 μM) were approximate.

### 2.6. Hepatoprotective Effects against Alcohol-Induced Damage

**Figure 10:**
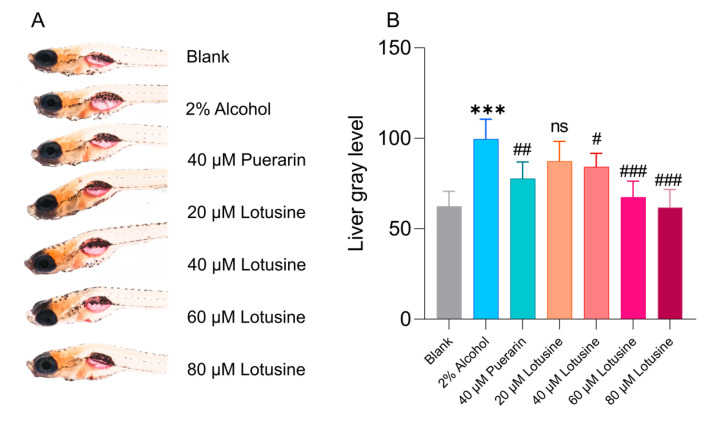
Alleviating effects of lotusine on hepatic steatosis induced by alcohol. (**A**) Photos of Oil Red O staining. (**B**) Quantitative analysis of results of Oil Red O staining. *** *p* < 0.001 vs. blank; ### *p* < 0.001 vs. alcohol exposure group; ## *p* < 0.01 vs. alcohol expose control; # *p* < 0.1 vs. alcohol expose control; ns, not significant; *n* = 7.

Alcoholic fatty liver disease can be induced in zebrafish larvae by exposing them to 2% alcohol. Oil red O is a fat-soluble azo dye that can label neutral lipids, which allows quantifying lipids in hepatic locations (Figure 10A). The larvae livers were stained over a larger area and darker after alcohol exposure. The stained photos were processed using ImageJ to quantify gray levels representing lipid levels. As shown in Figure 10B, the liver greyscale obviously increased to 110 in alcohol exposure compared with the blank control. When treated with puerarin, the gray level decreased to 68, which was approximate to the level (61) of the 80 μM lotusine-treated group.

### 2.7. Lotusine Toxicity Evaluation

**Figure 11:**
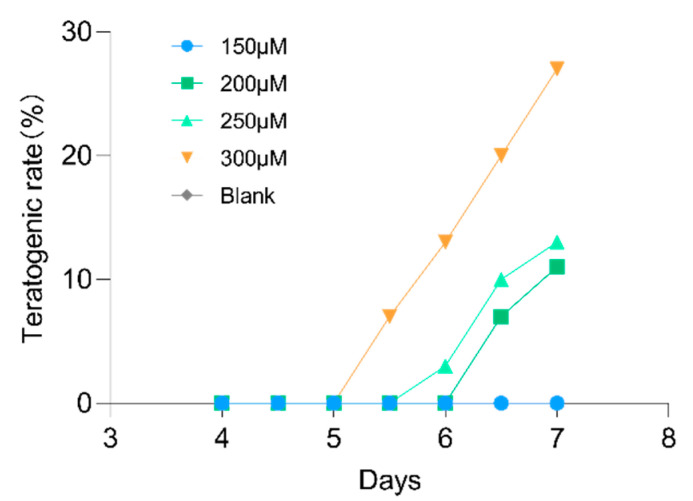
Developmental toxicity of lotusine to zebrafish larvae (*n* = 3, 30 larvae per group). No death appeared in blank controls.

Toxicity is a significant influence on the availability of an active molecule. Zebrafish larvae are susceptible to exogenous substances and are therefore commonly used to measure the toxicities of drugs or environmental pollutants. We used the teratogenicity rate to measure the toxicity of lotusine to zebrafish larvae. The larvae were exposed to a range concentrations of lotusine from 150 μM to 300 μM for 7 days. As shown in Figure 11, no zebrafish larvae developed malformations when exposed to lotusine below 200 μM throughout the 7 days. At the effective dose of lotusine (40 μM), it was safe enough to the larvae. Deformities were only observed in high concentration (>250 μM) exposure for more than 5 days. When zebrafish larvae were treated with 300 μM lotusine, the deformity rate rose to 27% on the 7th day of exposure. Lotusine caused 3% larvae death at 250 µM for 7 days. And the death rate went up to 9% at 300 μM lotusine for 7 days.

## 3. Discussion

### 3.1. The Scene of Alcohol-Induced Metabolic Disorder in Zebrafish Larvae

We selected zebrafish larvae as a model for in vivo metabolism research. Zebrafish metabolism is approximate to that of humans and mammals. The larvae are transparent and easy to observe. Zebrafish are generally administered by exposure, i.e., the drug or interesting compounds are dissolved in the culture water. Thus, in vivo metabolism was less susceptible to feeding time than oral administration in mice. Furthermore, the tiny size of the larvae body was highly suitable for bioassay for a trace amount of natural products. Therefore, to better assess the repairing effects of the two natural products (lotusine and puerarin) on the alcohol-damaged metabolome, we first explored the effects of alcohol on metabolism in zebrafish. Theoretically, different doses of alcohol would have varying degrees of impact on in vivo metabolism, but it was still unclear whether the extent of metabolic impairment responds to alcohol dose.

The differential metabolites induced by 1% and 2% alcohol exposure were very close. They shared 54 same differential metabolites. Although a high concentration of alcohol exposure (G2) elicited more 28 metabolites differences, the enrichment based on the G2/G0 and G1/G0 differential groups showed that they were both highly focused on pathways and modules related to liver disease and neurodegenerative diseases. Apoptosis and necroptosis are typically programmed cell death, which involves the liver and neurodegenerative diseases [33]. The pathways of fatty acid elongation and biosynthesis of unsaturated fatty acids are generally approached to fatty acid synthesis, which takes an essential role in the development of alcoholic liver disease. Nucleotide metabolism is usually involved in parasympathetic neuron growth [34] and DNA damage in the liver [35].

The enrichment results also added cases to demonstrate the proximity between zebrafish metabolism and that of mammals. It also showed that LC-MS based metabolic analysis can track alcohol-induced metabolism variation or damage. On this basis, we can furtherly explore the metabolic repair role of natural products or drugs.

### 3.2. Puerarin Repair against Alcohol-Induced Metabolism Disorder

Puerarin was reported to alleviate dysfunctional nerve damage and liver damage caused by alcohol consumption or abuse, which was often used as a positive reference for related bioassay studies [36,37]. Here we also selected puerarin as a positive control to explore the role of puerarin and lotusine from a metabolome perspective. As analyzed above, alcohol-induced metabolites were up-regulated or down-regulated. So here, we searched for metabolites whose trends were reversed when the larvae were treated with puerarin or lotusine. In the puerarin-treated group (G3), seven metabolites met this changing trend (Figure 5). The enriched pathways and modules and their relationship are summarized in Figure 6. Among the KEGG nodes, sphingolipid metabolism, fatty acid metabolism and elongation are highly related to lipid metabolism.

Among the enriched KEGG pathway and modules, necroptosis, sphingosine and fatty acid metabolism were highly related to liver damage and protection. Necroptosis is a significant feature of the alcoholic liver and plays a crucial role in alcohol-induced liver damage [38]. Alcohol exposure induces receptor-interacting protein kinase 1 (RIPK1), a signaling molecule activating necroptosis [39]. The related metabolite IMP (C00130) was down-regulated when larvae were exposed to alcohol and approximately up-regulated to a normal level by puerarin treatment. Sphingolipid and fatty acid metabolism contributed to non-alcoholic fatty liver disease [40]. Since sphingolipid contains long-chain saturated and unsaturated fatty acids, abnormal metabolism of fatty acids directly affects the sphingolipid (C00319) level. In our exploration, alcohol exposure led to down-regulating saturated fatty acid (icosanoic acid, C06425) and sphingolipid (C00319) levels, restored by puerarin.

Pathways of necroptosis and sphingolipid metabolism were also related to nerve damage and neuroprotection. Inhibiting necroptosis was considered a potential target for neuroprotection [41]. Sphingolipid metabolism was reported involvement in neurodegeneration [42]. Overall, the necroptosis and sphingolipid metabolism are hubs of the repainting network of puerarin. Both pathways played a dual role of liver protection and neuroprotective, which were confirmed by the behavioral evaluation and liver injury bioassay (Figure 9 and Figure 10). Thus, necroptosis and sphingolipid metabolism were the critical pathways for the dual action of puerarin.

### 3.3. Lotusine Repair against Alcohol-Induced Metabolism Disorder

Lotusine-regulating metabolites were quite different from that of puerarin, except for the steroid C05465. Lotusine restored the C05465 level better than puerarin. Moreover, lotusine increased unsaturated fatty acid C04805, which was in the pathway of glutathione metabolism, not on the fatty acid biosynthesis approach. The effect of lotusine on lipids metabolism was not more promising than that of puerarin. Another regulated metabolite, C00214, was involved in nucleotide metabolism. Not all differential metabolites were enriched into the KEGG signaling pathways. Such as the phosphoric acid derivative C04234 and steroid C08158 have no pathway information in the KEGG database since their physiological roles and pathways information are still uncertain.

Lotusine was first reported to have a mitigating effect on alcohol damage. Phenotypic bioassays showed that this compound had hepato- and neuroprotective effects comparable to puerarin. The reversed metabolites and enriched pathways revealed that lotusine regulated different metabolic pathways than puerarin (Figure 8). The enriched pathway network showed two clusters, one centered on glutathione and the other on the tightly connected pathways of pyrimidine and nucleotide. Hepatic glutathione metabolism was generally decreased to meet the increased oxidant requirements after alcohol exposure [43]. Moreover, glutathione was also a major antioxidant in the brain. It acted as an electron donor to scavenge peroxides via non-enzymatic pathways and as a neurotransmitter to modulate oxidative responses [44]. The glutathione response to lotusine repairing was also involved in an oxidation module (beta-oxidation, M00862) in the primary bile acid pathway. In our observations, the glutathione-related metabolites 5-HETE (C04805) and taurochenodeoxycholate (C05465) were decreased by alcohol exposure and were restored by lotusine treatment. Nucleotide and pyrimidine were on a different network cluster from the glutathione pathway. Nucleotide metabolism was related to parasympathetic neuron growth [34] and DNA damage in the liver [35]. Metabolic repair did not independently correspond to phenotypic activities. From our metabolic perspective, the dual role of lotusine encompassed oxidative regulation involving glutathione as well as neuronal growth and DNA repair involving nucleotide metabolism.

### 3.4. Neuroprotection and Hepatoprotection of Puerarin and Lostusine

Metabolic analysis showed that puerarin and lotusine could antagonize alcohol-induced metabolic disorders. This restorative action suggested a correlation between fatty liver development and nerve cell apoptosis. In the phenotype observation, we aimed to compare the neuro- and hepatoprotectiion of puerarin and lotusine. According to the metabolic exploration, neuroprotective function was mainly related to necroptosis and nucleotide metabolism, while hepatoprotection was related to fatty acid biosynthesis and metabolism. Puerarin had already been verified as a potential agent against alcoholism injury. Therefore, puerarin was used as a positive control to compare the protections between it and lotusine.

In behavioral observations, we used 5 dpf (days post fertilization) larvae since the central nervous system of larvae becomes fully developed at this time. Alcohol exposure of 1% can cause larvae to be more active. The move distance and velocity were significantly higher than blank controls. Puerarin and lotusine significantly recovered abnormal behavior, but the behavior was not fully restored to normal levels, which might be due to the persistent exposure to alcohol during observation. The neuroprotective effect of lotusine also showed a dose correlation. Both compounds (puerarin and lotusine) have a potential activity to protect nerve cells from apoptosis even to normal levels. Lotusine showed slightly more neuroprotection than puerarin. Puerarin showed slightly superior activity against lipids accumulation in the liver, possibly due to regulating fatty acid biosynthesis and metabolism. Lotusine is a compound of food material origin and is highly safe, even though no mortality was observed in zebrafish exposed to 300 μM of lotusine within 5 days. The compound possesses enough safe space for further development of functional foods.

### 3.5. Limitations

Widely targeted metabolomics analysis can only rely on identified metabolites for bioinformatics analysis. Due to chromatographic separation and the reference MS/MS library’s size, many metabolites still cannot be accurately identified. Thus, it is necessary to expand the reference library continuously to reduce the dark region in metabolome detection. Moreover, the low content of lotusine in *Nelumbinis plumula* also limits further development. Therefore, it is necessary to look for similar alternative compounds or other plant resources containing this ingredient.

## 4. Materials and Methods

### 4.1. Materials and Chemicals

Analytically pure mineral salts (CaCl_2_, MgSO_4_, KCl, NaCl) and phosphate buffers were purchased from Solarbio Science & Technology Co., Ltd. (Beijing, China). Lotusine and puerarin were purchased from PureChem-Standard Co., Ltd. (Chengdu, China). AB wild adult zebrafish were purchased from FishBio Co., Ltd. (Shanghai, China).

### 4.2. Zebrafish Maintaince and Embryo Collection

Adult female and male zebrafish were maintained in 28 ± 0.5 °C fresh water containing 145 mg/L sea salt. We randomly selected zebrafish females and males (3:2) and separately placed them in the spawning tanks at 9:00 p.m. The partitions between male and female fish were removed in the following 8:00 a.m. The males chased the females and began naturally spawning. We collected embryos at 12:00 a.m. and maintained them in freshly prepared E3 water. The maintenance E3 was replaced twice daily. Dead eggs (white eggs) were picked out before E3 water replacement.

### 4.3. Preparation of Metablic Samples

We set up five parallel groups: blank control (G1), 1% alcohol exposure group (G1), 2% exposure group (G2), 40 µM exposure group (G3) and 40 µM lotusine exposure group (G5). Zebrafish larvae were maintained in E3 water without other treatments. G1 and G2 groups were exposed to 1% and 2% alcohol for 36 h in E3 water from 5 dpf till collection. G3 and G5 groups were added 40 µM puerarin and lotusine respectively on 4 dpf, then on 5 dpf were exposed in 2% alcohol for 36 h before being collected. All cultured water were changed twice daily. Each group was conducted with 7 replicates and 50 larvae per replicate.

Each collected replicate sample was washed with fresh pure water and transferred into a 1.5 ml centrifuge tube to rapidly freeze with liquid N_2_. Metabolites of each sample was extracted with 350 μL MeOH for 4 min in a JY92-IIDN cell disruptor (Ningbo Scientz Biotechnology Co., Ltd., Ningbo, China). The ultrasonic power of the disruptor was set to 15%. All operations were performed on an ice surface or in an ice water mixture. Then, all samples were centrifuged at 4 °C, and the supernatant was filtered through a syringe filter membrane (0.22 μm) before LC-MS/MS analysis.

### 4.4. LC-MS/MS Analysis

Metabolites were analyzed on a Q Exactive Focus instrument (Thermos Fisher Scientific, Waltham, MA, USA) equipped with a Vanquish UPLC. The metabolites were chromatographed on an Accucore aQ C18 column (150 × 2.1 mm, 2.6 µm, Thermos Fisher Scientific, Waltham, MA, USA). The mobile phase was 2% MeCN (containing 0.1% AcOH) for the first 2 min, then increased to 50% at 5 min, to 100% at 15 min, and then kept 100% for 2 min. Mass spectra were acquired using Full MS-AIF mode with resolutions 70,000. Ions were scanned from 70 to 1,000 *m/z*. AGC target was set to 1.0 × 10^6^. The collision energy was set to 10, 20, 40 V for AIF. The MS raw data were recorded in profile type.

### 4.5. LC-MS Data Analysis

The ESI positive and negative raw files were separately processed using MS-Dial version 4.90 [45]. After the raw data files were imported into MS-dial, MS1 and MS2 tolerance in centroid process were respectively set to 0.01 and 0.05. Peaks were detected with the minimum thresholds (width 5, height 2 × 10^6^) and smoothed by the Linear Weighted Moving Average (smoothing level 3). Deconvolution was performed with a Sigma window value 0.5. We used KEGG compound database as blueprint to create a reference MSP database, which integrated experimental HMDB (5.0) MSMS spectra [46].and CFM-ID (version 4.0) predicted MSMS spectra [47,48]. In the metabolite identification process, accurate mass tolerances were set to 2 mDa for MS1 and 5 mDa for MS2. QC data were selected as alignment reference. The result alignment data were normalized by the LOWESS method (span 0.6) based on QC control. Then the normalized data were exported after zero values were replaced with 1/10 of minimum peak height over all samples. The exported positive and negative ions data were combined using R scripts and performed PCA and sPLD-DA analysis using Bioconductor R package mixOmics [49]. The differential metabolites (DMs) were filtered with the threshold of FC > 1.5 and Student’s *t*-test *p*-value < 0.01. We selected the differential metabolites to enrich pathway using the Bioconductor package FELLA (1.16.0) [50] with *p*-score threshold of 0.01.

### 4.6. Evaluation of Neuronal Apoptosis

Apoptotic neurons in zebrafish larvae were induced following a reported procedure [51]. In brief, the 4-dpf zebrafish larvae were treated with puerarin (40 μM) or lotusine (20 μM, 40 μM, 60 μM and 80 μM) for 24 h. Then the larvae were exposed to 1% alcohol in E3 water for 36 h. Then, the larvae were washed twice with PBS and incubated with 5 mg/L acridine orange (AO) in the dark for 30 min. After being washed three times with PBS, they were anesthetized using 0.016% tricaine in culture water. We used Nikon fluorescence stereoscope smz25 (Nikon Corp, Tokyo, Japan) to observe the apoptotic nerve cells.

### 4.7. Zebrafish Locomotor Behavior Measurements

The 5 dpf zebrafish larvae were treated with puerarin (40 μM) or lotusine (20 μM, 40 μM, 60 μM and 80 μM) for 24 h. We placed each group of zebrafish in a 6-well plate and then added 1% alcohol. After adapting for 10 min, the larvae were recorded for half an hour using an electron microscope of BC4K-36D2 model (Dongwan Gopoint Co., Ltd, Guangdong, China). Zebrafish larvae were euthanized with tricaine after observation according to the requirements of animal experiment ethics. Their motion trajectory was analyzed using Ethovision software (Noldus Information Technology bv., Wageningen, The Netherlands).

### 4.8. Evaluation of Alcohol-Induced Fatty Liver

The evaluation was parallelly performed on the groups of blank control, alcohol exposure group, puerarin- or lotusine-treated groups. In the alcohol exposure group, the 5 dpf larvae were exposed to 2% alcohol for 36 h in E3 water before being staining observation. In puerarin- or lotusine-treated groups, the larvae were firstly treated with puerarin (40 µM) or lotusine (20, 40, 60 and 80 μM, respectively) on 4 dpf, then exposed on 5 dpf in 2% alcohol for 36 h.

After that, each larva was stained with 0.5% Oil Red O (in 60% isopropanol) as previously described [18]. The larvae were washed three times with PBS, fixed with 4% paraformaldehyde for 4 h, washed twice with PBS and then immersed in 60% propylene glycol for 20 min. Then they were stained with Oil Red O dye for three hours in the dark and washed with 60% propylene glycol and PBS. The lipid droplets in the liver were observed and photographed with Nikon fluorescence stereomicroscope SMZ25 (Nikon Corp, Tokyo, Japan). The photos were converted to grayscale and quantified by ImageJ.

### 4.9. Lotusine Toxicity Evaluation

To investigate the developmental toxicity of lotusine, we transferred 8 h post-fertilization (hpf) embryos into six-well plates. To each well was added 30 embryos and 3.00 mL E3 water containing lotusine (0, 150, 200, 250 or 300 μM). Each concentration of lotusine was conducted with three replicates and treated for 7 days. The E3 water was changed every 24 h. Teratogenicity and mortality rate were counted every 12 h.

## 5. Conclusions

We selected zebrafish larvae as a sensitive platform for mining metabolic response to alcoholism and compounds with repair functions on metabolic dysfunction. Alcoholism can invoke numerous metabolic imbalances. The most obvious were apoptosis, necroptosis, fatty acid biosynthesis and nucleotide metabolism. The hepatoprotective molecule puerarin was found to correct metabolic abnormalities through sphingolipid metabolism and necroptosis approaches. Lotusine is a newly discovered alkaloid with mitigating effects on alcohol damage. The alkaloid can reverse metabolic disorders in nucleotide and glutathione metabolism. Both compounds can relieve abnormal behavior and neuron apoptosis induced by alcohol. And they also showed hepatoprotective by reducing lipid accumulation in the liver. The present study newly found lotusine repair on metabolic damage invoked by alcohol and elucidated its role in restoring abnormal metabolism.

## Figures and Tables

**Table 1:** Enrichment of differential metabolites invoked by alcohol (G2/G0 and G1/G0).

KEGG ID	KEGG Name	−log (*p*) G2/G0	−log (*p*) G1/G0
map00062	Fatty acid elongation	6.00	4.19
map04210	Apoptosis	6.00	6.00
map04216	Ferroptosis	6.00	Not hit
map04217	Necroptosis	6.00	6.00
map04310	Wnt signaling pathway	6.00	Not hit
map03013	Nucleocytoplasmic transport	5.28	Not hit
map01040	Biosynthesis of unsaturated fatty acids	4.95	2.7
map01232	Nucleotide metabolism	3.81	5.65
map04080	Neuroactive ligand-receptor interaction	3.03	Not hit
map00730	Thiamine metabolism	2.67	4.21
map04540	Gap junction	2.56	Not hit
map03320	PPAR signaling pathway	2.09	Not hit
map03410	Base excision repair	Not hit	2.33
M00099	Sphingosine biosynthesis	4.56	6.00
M00958	Adenine ribonucleotide degradation, AMP => Urate	4.20	6.00
M00094	ceramide biosynthesis	4.06	6.00
M00959	Guanine ribonucleotide degradation, GMP => Urate	3.32	5.15
M00042	Catecholamine biosynthesis, tyrosine => dopamine => noradrenaline => adrenaline	2.83	Not hit
M00892	UDP-N-acetyl-D-glucosamine biosynthesis, eukaryotes, glucose => UDP-GlcNAc	2.48	Not hit
M00096	C5 isoprenoid biosynthesis, non-mevalonate pathway	Not hit	3.44
M00170	C4-dicarboxylic acid cycle, phosphoenolpyruvate carboxykinase type	Not hit	2.69
M00014	Glucuronate pathway (uronate pathway)	Not hit	2.48

## Data Availability

The raw data are available on request from the corresponding author.

## Supplementary Materials

The following supporting information can be downloaded at: https://www.mdpi.com/article/10.3390/ijms231810385/s1.
